# A Case of TAFRO Syndrome Developed after COVID-19 Vaccination

**DOI:** 10.1155/2023/7292895

**Published:** 2023-12-04

**Authors:** Hitomi Hirose, Hitoshi Suzuki, Yukako Umezawa, Masako Iwasaki, Hiromitsu Fukuda, Hisatsugu Takahara, Shigeki Tomita, Yusuke Suzuki

**Affiliations:** ^1^Department of Nephrology, Juntendo University Urayasu Hospital, Chiba, Japan; ^2^Department of Nephrology, Juntendo University Faculty of Medicine, Tokyo, Japan; ^3^Department of Pathology, Juntendo University Urayasu Hospital, Chiba, Japan

## Abstract

TAFRO syndrome is a systemic inflammatory disorder, which is characterized by thrombocytopenia, anasarca, fever, reticulin myelofibrosis, renal dysfunction, and organomegaly. It often presents with progressive clinical symptoms and can be fatal. COVID-19 vaccination is important to reduce the number of COVID-19-infected populations and lower the risk of becoming severe. However, serious adverse events have been reported. TAFRO syndrome that progresses after the COVID-19 mRNA vaccination has not yet been reported. A 45-year-old man developed fever, gross hematuria, renal dysfunction, pleural effusions, and ascites immediately after vaccination. This case fulfilled three major categories (thrombocytopenia, anasarca, and systemic inflammation) and two minor categories (renal insufficiency and myelofibrosis) and was diagnosed with TAFRO syndrome. High-dose steroid treatment was initiated, followed by prednisolone administration. After treatment, renal dysfunction and fluid retention were resolved. Universal vaccination against COVID-19 is important for lowering the risk of spreading COVID-19 infection. Several complications, such as renal, hematological, and heart diseases, have been reported; however, its pathogenesis is unclear. The possibility of various complications after the COVID-19 vaccination, including TAFRO syndrome, should be considered.

## 1. Introduction

The TAFRO syndrome, a rare systemic disease characterized by thrombocytopenia, anasarca, fever, reticulin myelofibrosis, renal dysfunction, and organomegaly, was first reported in Japan in 2010. The diagnostic criteria for TAFRO syndrome were determined by the All Japan TAFRO Syndrome Research Group in the Research Program for Intractable Disease by the Ministry of Health, Labor, and Welfare Japan in 2015 and updated in 2019 [1, 2].

Castleman's disease (CD) is classified into unicentric CD (UCD) and multicentric CD (MCD), and MCD has three subtypes: POEMS syndrome, HHV-8-associated MCD, and idiopathic MCD (iMCD) [3]. Several clinical and pathological characteristics of TAFRO syndrome resemble those of iMCD. TAFRO syndrome is distinct from POEMS (polyneuropathy, organomegaly, endocrinopathy, monoclonal plasma cell proliferation, and skin change) syndrome or iMCD-not otherwise specified (iMCD-NOS), as it does not accompany overproduction of immunoglobulin or polyneuropathy. Fujimoto S. et al. reported that iMCD can be classified into two distinct subtypes: TAFRO-iMCD and iMCD-NOS [4]. Although the concepts of iMCD and TAFRO syndrome may overlap, TAFRO syndrome often presents with progressive clinical symptoms and can be fatal.

COVID-19 vaccination is recommended to reduce the number of COVID-19-infected population and lower the risk of becoming severe. However, some serious adverse events have been reported after the COVID-19 vaccination, such as anaphylaxis, thrombosis, Guillain–Barre syndrome, myocarditis, and pericarditis [5, 6]. Although these complications are not frequent, these incidents may lead people to avoid vaccination and make the pandemic to the worse.

Here, we report a case of TAFRO syndrome that developed after the first injection of a COVID-19 mRNA vaccine.

## 2. Case Presentation

A 45-year-old man was transferred to our hospital for the evaluation of renal dysfunction and anasarca. After the first dose of the COVID-19 mRNA vaccine (Moderna mRNA-1273), he developed fever and macrohematuria for 3 to 4 days and developed edema and abdominal distension from the day after the first COVID-19 mRNA vaccination. Six days after vaccination, his symptom was persisted and he was detected renal dysfunction (eGFR 39.2 mL/min/1.73 m^2^, serum creatinine 1.59 mg/dL), elevated C-reactive protein (22.4 mg/dL), and proteinuria. The patient has a history of atopic dermatitis. It was the first time COVID-19 vaccination for him, and there were no adverse reactions or allergies caused by the other vaccines.

On admission to our hospital, the patient was 171.0 cm tall and weighed 80.0 kg (weight gain of 7 kg in 1 week), with a blood pressure of 142/73 mmHg, a pulse rate of 60/min, and a body temperature of 37.3 degrees. He complained of epigastric pain, dyspnea, and bilateral pitting edema of the lower extremities. Oxygen saturation was 92% in room air. Electrocardiography was normal, and an echocardiogram revealed normal left ventricular systolic function. Laboratory data revealed elevated levels of C-reactive protein (22.6 mg/dL) and renal dysfunction (eGFR 49 ml/min/1.73 m^2^). The platelet count was 9.1 × 10^3^/*μ*L. Examination of antinuclear antibody, antidouble-stranded DNA IgG antibody, antineutrophil cytoplasmic antibody, and antiglomerular basement membrane antibody was all negative. The patient has a history of atopic dermatitis and indicates high level of serum IgE (Table 1). The PCR test results for COVID-19 were negative. Procalcitonin was slightly high at 2.66 ng/mL; however, antibiotics did not effective to reduce CRP during the clinical course. Physical findings showed no signs of infection.

**Figure 1:**
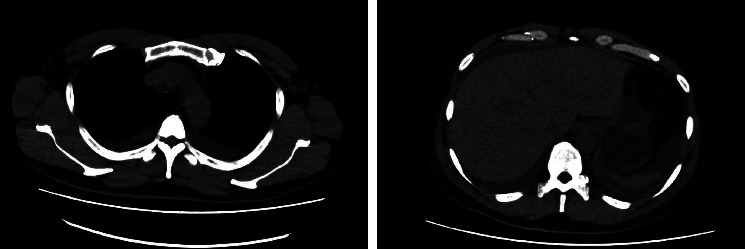
Computer tomography showed bilateral pleural effusions and ascites but no organomegaly.

Bilateral pleural effusions and a slight accumulation of ascites were detected on computed tomography (CT) (Figure 1). Small lymph nodes in the axilla were detected on CT; however, they were too small to perform biopsy. There was no abnormal uptake on gallium scintigraphy.

Levels of interleukin-6 (IL-6) and vascular endothelial growth factor (VEGF) in pleural effusions and ascites were significantly elevated. However, carcinoembryonic antigen (CEA) in pleural effusion and ascites were negative (Table 2).

Bone marrow biopsy showed hyperplastic and increased megakaryocytes, and reticular fiber hyperplasia was partially observed by silver staining. The extent of myelofibrosis was equivalent to MF1-2.

**Figure 2:**
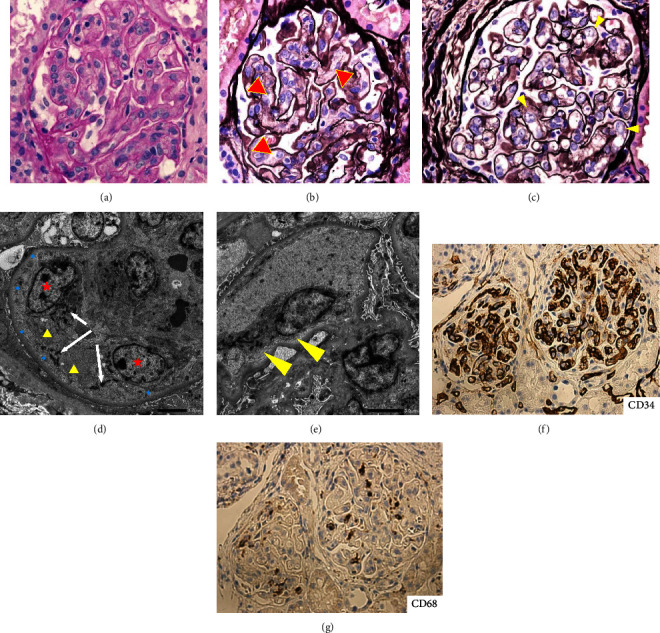
Renal histological analysis. (a) There were diffuse and global endothelial cell enlargements by cytoplasmic swelling with large numbers of inflammatory cells (PAS staining, ×400). (b) Mesangiolysis. Partial dissolution of the mesangial matrix (arrowhead) (PAM staining, ×400). (c) Endothelial cell swelling (arrowhead) (PAM staining, ×400). (d) Endothelial cell swelling occluding the capillary lumen (star) with loss of fenestrations (triangle), and expansion of the subendothelial space (diamond). Fibrin deposition was observed (white arrow). (e) Expansion of the subendothelial space was observed. Immunoperoxidase staining for CD34 (f) and CD68 (g) was positive.

We performed a renal biopsy for the definitive diagnosis of renal injuries. Light microscopy showed diffuse hypercellularity with thrombotic microangiopathy (TMA) lesions of the glomeruli. There was diffuse and global endothelial cell enlargement due to cytoplasmic swelling, with a large number of inflammatory cells (Figure 2(a)). The partial dissolution of the mesangial matrix (mesangiolysis) is also shown (Figure 2(b)). Endothelial cell swelling occluding the capillary lumen with loss of fenestration and expansion of the subendothelial space was observed by electron microscopy (Figures 2(d) and 2(e)). Immunoperoxidase staining for CD34 and CD68 were positive (Figures 2(f) and 2(g)). Immunofluorescence analysis revealed negative staining for IgG, IgA, IgM, C3, and C1q.

Overall, this patient fulfilled three major categories (thrombocytopenia, anasarca, and systemic inflammation) and two minor categories (renal insufficiency and myelofibrosis). Thus, the patient was diagnosed with TAFRO syndrome by diagnostic criteria for TAFRO syndrome [1, 2]. After a second high-dose steroid pulse therapy with 500 mg methylprednisolone for 3 days, followed by prednisolone (PSL) 40 mg/day, anasarca, systemic inflammation, and renal injuries were improved (C-reactive protein <0.1 mg/dL, eGFR 67 ml/min/1.73 m^2^). The platelet count increased to the normal range on the 31st day of admission. Eleven months after discharge, the patient had never relapsed under PSL treatment (5 mg/day).

## 3. Discussion

Several side effects of COVID-19 vaccination have been reported worldwide. Patone et al. reported myocarditis, pericarditis, and cardiac arrhythmias after the mRNA COVID-19 vaccination [6]. Cases of kidney injury, such as IgA nephropathy [7], minimal change disease [8–10], and IgG4-related disease [11], following mRNA vaccination has accumulated in current research. Recently, few cases of TAFRO syndrome [12, 13] and iMCD [14] developed after COVID-19 vaccination or COVID-19 infection was reported. Yamada et al. reported TAFRO syndrome with a serious clinical course triggered by the second COVID-19 mRNA vaccination [12]. Hoffman et al. reported a case of iMCD triggered by the second COVID-19 mRNA vaccination [14]. We encountered a case of TAFRO syndrome that developed immediately following the first COVID-19 mRNA vaccination. Most vaccine-related disease onsets were associated with the COVID-19 mRNA vaccine.

Mizuno et al. reported the renal histology of TAFRO syndrome as a glomerular endotheliopathy that is representative of endothelial cell swelling, mesangiolysis, mesangial loosening (loss of mesangial matrix staining), and GBM double contour and thickening. Electron microscopy shows loss of mesangial architecture and endothelial space, as well as loss of endothelial cell fenestration [15]. In the present case, these findings, except for endothelial cell fenestrations, were detected, confirming the diagnosis of TAFRO syndrome.

Previous reports have suggested that vaccination may trigger an autoimmune response due to antigenic mimicry as well as the activation of quiescent autoreactive T and B cells [16]. These reports suggest that the COVID-19 mRNA vaccine stimulates the immune system and causes autoimmune reactions.

Although the etiology of TAFRO syndrome has not been revealed, it has been demonstrated that hypercytokinemia related to IL-6 and stimulation of VEGF contributes to the pathophysiological mechanisms [17, 18]. It is suggested that the abnormal autoimmune response induced by COVID-19 mRNA vaccination caused the overproduction of cytokines, such as IL-6 and VEGF, in the present case. As treatments for TAFRO syndrome, corticosteroid is selected as the first line. Other immunosuppressive drugs such as tocilizumab and rituximab are used in combination in case with relapse or steroid-refractory course [18]. In present case, corticosteroid therapy was effective, and renal dysfunction and fluid retention resolved.

The pathophysiology of TAFRO syndrome has not yet been clarified; however, it often presents with progressive clinical symptoms and can be fatal. We should be considered as one of the rare side reactions of the COVID-19 vaccine and should be diagnosed earlier. Further studies are needed to reveal the mechanisms of the immune response in developing severe side effects following the COVID-19 mRNA vaccination.

## 4. Conclusion

Universal vaccination against COVID-19 is important for lowering the risk of spreading COVID-19 infection. Several complications, such as renal, hematological, and heart diseases, have been reported; however, its pathogenesis is unclear. The possibility of various complications after the COVID-19 vaccination, including TAFRO syndrome, should be considered.

## Figures and Tables

**Table 1:** Laboratory data on admission.

Complete blood cell count	Reference range	
White blood cell (/*μ*L)	19,000	(4,000∼8,000)
Neutrophil (%)	87.4	(45∼60)
Eosinophil (%)	0.2	(1∼5)
Lymphocyte (%)	4.3	(25∼45)
Hemoglobin (g/dL)	13.7	(14∼18)
Platelet (×10^4^/*μ*L)	9.1	(15∼35)

*Coagulation test*		
APTT (s)	33.2	(30.2)
PT (%)	77	(70∼100)
PT-INR	1.19	(0.9∼1.1)
D-dimer (*μ*g/mL)	25.58	(0∼1)
Fibrinogen (mg/dL)	506	(150∼400)
TAT (ng/mL)	4.3	(0∼3)

*Blood chemistry*		
Albumin (g/dL)	1.9	(3.9∼4.9)
AST (IU/L)	16	(13∼33)
ALT (IU/L)	14	(8∼42)
LDH (IU/L)	309	(124∼222)
CPK (IU/L)	66	(60∼287)
ALP (IU/L)	111	(38∼113)
ɤ-GT (IU/L)	44	(10∼47)
UN (mg/dL)	31	(8∼22)
Creatinine (mg/dL)	1.29	(0.61∼1.04)
Urinary acid (mg/dL)	9.2	(2∼7)
Na (mmol/L)	141	(138∼146)
K (mmol/L)	3.8	(3.6∼4.9)
Cl (mmol/L)	108	(99∼109)
IgG (mg/dL)	602	(870∼1700)
IgA (mg/dL)	158	(110∼410)
IgM (mg/dL)	25	(35∼220)
IgE (mg/dL)	1759.0	(0∼232)
C3 (mg/dL)	141	(65∼135)
C4 (mg/dL)	34	(13∼35)
CH50 (U/mL)	69	(32∼49)
C-reactive protein (mg/dL)	22.6	(0∼0.3)
BNP (pg/mL)	248.7	(0∼18.4)
IL-6 (pg/mL)	17.2	(0∼4)
ANA	<40	(0∼40)
ds-DNA (U/mL)	<1.0	(0∼10)
MPO-ANCA (U/mL)	<1.0	(0∼3.5)
PR3-ANCA (U/mL)	<1.0	(0∼3.5)
GBM (U/mL)	<1.0	(0∼3)

*Urinalysis*		
Sediment		
RBC (/HPF)	20–29	<1–4
WBC (/HPF)	10–19	<1–4
Cast		
Granule cylinder	1+	
Tubular epithelium	1+	

*Urinalysis*		
Protein (g/gCre)	0.67	<0.15
NAG (IU/L)	120.5	(0.97∼4.17)
*β*2 microglobulin (*μ*g/L)	22,500	(0∼230)

APTT; activated partial thromboplastin time, PT; prothrombin time, TAT; thrombin-antithrombin complex, AST; aspartate aminotransferase, ALT; alanine aminotransferase, LDH; lactate dehydrogenase, CPK; creatine phosphokinase, ALP; alkaline phosphatase, BUN; urea nitrogen, Na; sodium, K; potassium, Cl; chlorine, IgG; immunoglobulin G, IgA; immunoglobulin A, IgM; immunoglobulin M, IgE; immunoglobulin E, BNP; brain natriuretic peptide, IL-6; interleukin-6, ANA; antinuclear antibody, ds-DNA; antidouble-stranded DNA IgG antibody, MPO-ANCA; perinuclear antineutrophil cytoplasmic antibody, PR3-ANCA; proteinase-3 antineutrophil cytoplasmic antibody, anti-GBM antibody; antiglomerular basement membrane antibody, RBC; red blood cells; WBC; white blood cells, NAG; N-acetyl-*β*-d-glucosaminidase.

**Table 2:** Analysis of pleural effusion and ascites.

Pleural effusions	Reference range	
sIL2-R (IU/mL)	2130	(157∼474)
IgG (mg/dL)	371	(870∼1700)
IgA (mg/dL)	78	(110∼410)
IgM (mg/dL)	12	(33∼190)
IL-6 (pg/mL)	707	(0∼4)
VEGF (pg/mL)	83	(0∼38.3)
CEA (ng/mL)	<0.5	(0∼5)

*Ascites*		
sIL2-R (IU/mL)	1580	(157∼474)
IgG (mg/dL)	247	(870∼1700)
IgA (mg/dL)	49	(110∼410)
IgM (mg/dL)	7	(33∼190)
IL-6 (pg/mL)	249	(0∼4)
VEGF (pg/mL)	129	(0∼38.3)
CEA (ng/mL)	<0.5	(0∼5)

sIL2-R; serum soluble interleukin-2 receptor, IgG; immunoglobulin G, IgA; immunoglobulin A, IgM; immunoglobulin M, IL-6; interleukin-6, VEGF; vascular endothelial growth factor, CEA; carcinoembryonic antigen.

## Data Availability

The datasets used in the current study are available from the corresponding author upon reasonable request.
